# The Administration of Anæsthetics

**Published:** 1907-07-20

**Authors:** 


### Resident Medical Officers' Department.

[Contributions to this Column are invited, and, if accepted, will be paid for.]

### THE ADMINISTRATION OF ANÆSTHETICS.

"Enquirer, M.D.," writes:—In your recent useful criticisms in The Hospital, on remarks made by Dr. Waldo, Coroner for the City and Southwark, with regard to enquiries held by him on fifty-five sudden deaths which occurred during anæsthesia, you quote Dr. Hewitt as saying: "It is doubtful whether there is any real advantage in practice in employing chloroform prepared from rectified spirit. The author has used methylated chloroform for many years, and cannot satisfy himself that it is inferior to ethyl alcohol chloroform. He has also used acetone-made chloroform with equally good results. He is forced to believe, indeed, that there is little or no substantial foundation for much that has been written concerning the importance of employing this or that brand."

I should like to point out that other authorities do not agree with this view of the matter. For instance, Mr. Edmund Boyle, anæsthetist to St. Bartholomew's Hospital, says, under date April 1907:—"Chloroform may be prepared from ethylic alcohol, methylated alcohol, or acetone. It is stated by some observers that these different varieties of the drug produce precisely similar effects. The majority of anæsthetists, however, prefer to employ ethylic chloroform, and believe it to be the safest preparation. This opinion is substantiated by experiments. Some animals are extremely susceptible to chloroform, and are readily poisoned by its vapour. A far greater percentage die under the administration of methylated chloroform than under that of ethylic chloroform, although the same care is exercised in each case."

Mr. Colt, Senior Resident Anæsthetist to St. Bartholomew's Hospital, in his evidence before Dr. Waldo, agrees with this view, and thinks it of advantage to the patient that, save in urgent cases, the responsibility of giving anæsthetics should alone rest with those on the hospital staff, who are specially qualified and appointed for the particular purpose.

With this view Mr. Thompson, Visiting Anæsthetist and Demonstrator of Anatomy at Guy's Hospital, does not agree.

It is, however, of interest to note that Dr. Hewitt, in the preface to his work already referred to, appears to agree with Dr. Waldo when he says:—"Another reform needed is the appointment within our hospitals of an adequate number of officers for the special administration of anæsthetics, the senior of such officers being a member of the staff and possessing academic or professional qualifications similar in every respect to those required for other staff appointments at his particular hospital. This special department of practice should, in fact, be expanded in such a way that every anæsthetic within our hospitals is given by an officer appointed for such duties. This would, of course, increase the number of junior hospital appointments, but it would be of great advantage, not only to the public, but to the profession, to have a constant stream of experienced anæsthetists issuing from our great centres of medical education. The present requirements of surgery, so far as the administration of anæsthetics is concerned, are of a totally different order from those which prevailed twenty or thirty years ago, when abdominal operations were in their infancy; and to meet such requirements the modern anæsthetist has come into existence. This product of recent times is not only able to obviate a considerable number of fatalities which would otherwise occur from the unskilled use of anæsthetics, but by adjusting his administrations so as to prevent surgical shock, by guarding against certain after-effects, and by other means, he is able to contribute in no small measure to the success of operations in general. . . . . . Yet it often happens, not only in hospital but in private practice, that the safety of a patient during perhaps the most critical hour of his life is entrusted to the mercies of one who has had little, if any, scientific training, and whose acquaintance with the powerful drugs he is handling bears an inverse ratio to the self-confidence displayed in their administration. This state of things is open to much criticism, and should certainly be remedied. Until some reforms of the kind here indicated have been introduced human life will continue to be sacrificed to an unnecessary extent, and the progress of surgery will be unnecessarily retarded."

